# Cluster-Fault Tolerant Routing in a Torus

**DOI:** 10.3390/s20113286

**Published:** 2020-06-09

**Authors:** Antoine Bossard, Keiichi Kaneko

**Affiliations:** 1 Graduate School of Science, Kanagawa University, Kanagawa 259-1293, Japan; 2 Institute of Engineering, Tokyo University of Agriculture and Technology, Tokyo 184-8588, Japan; k1kaneko@cc.tuat.ac.jp

**Keywords:** fault tolerance, sensor, IoT, algorithm, information dissemination, interconnection network

## Abstract

The number of Internet-connected devices grows very rapidly, with even fears of running out of available IP addresses. It is clear that the number of sensors follows this trend, thus inducing large sensor networks. It is insightful to make the comparison with the huge number of processors of modern supercomputers. In such large networks, the problem of node faults necessarily arises, with faults often happening in clusters. The tolerance to faults, and especially cluster faults, is thus critical. Furthermore, thanks to its advantageous topological properties, the torus interconnection network has been adopted by the major supercomputer manufacturers of the recent years, thus proving its applicability. Acknowledging and embracing these two technological and industrial aspects, we propose in this paper a node-to-node routing algorithm in an n-dimensional k-ary torus that is tolerant to faults. Not only is this algorithm tolerant to faulty nodes, it also tolerates faulty node clusters. The described algorithm selects a fault-free path of length at most n(2k+⌊k/2⌋−2) with an $O(n^{2}k^{2}|F|)$ worst-case time complexity with F the set of faulty nodes induced by the faulty clusters.

## 1. Introduction

As mentioned, for instance, in [1,2], the number of Internet-connected devices is seeing a very rapid growth, with even fears of running out of available IP addresses. It is clear that the number of sensors follows this trend, thus inducing large sensor networks. The interconnection issue of sensor networks is thus critical. Given the large number of sensors involved, it is critical that these interconnection networks come with efficient data communication algorithms to maximise the performance of the sensor network. Because hardware failure is highly probable given the scale of the network, the tolerance to faults by such routing algorithms is key to data communication efficiency and robustness.

Considering the number of network nodes involved, it is insightful to make the comparison with the number of processors of modern supercomputers. In such large networks of processors or sensors, the problem of faulty nodes necessarily arises, with faults often happening in clusters. The tolerance to faults, and especially cluster faults, is thus critical as detailed below. Featuring advantageous topological properties, the torus network [3] has become very popular as interconnection network of supercomputers (e.g., IBM Blue Gene/L and Blue Gene/P, Cray Titan (Gemini interconnect [4]) and Fujitsu K (Tofu interconnect [5])) [6], thus proving the applicability of the torus topology for large network interconnection. Such topological properties of an *n*-dimensional *k*-ary torus include the network degree 2n, the diameter n⌊k/2⌋ and the network order (i.e., number of nodes) $k^{n}$. This is to be compared with, for instance, the hypercube network [7] (*n*-cube) that was favoured for earlier supercomputers (e.g., the Cosmic cube [8]), of degree and diameter *n* and network order $2^{n}$. So, given the physical restrictions on the number of links per node, that is, on the network degree, a torus is able to connect far more nodes than a hypercube: a small dimension *n* keeps the degree low, while its arity *k* can be adjusted to fit the number of nodes. The formal definition of a torus is given in the next section. Tori are also used for hierarchical interconnection networks [9].

From Menger’s theorem [10], node-to-node routing can tolerate at most d−1 faulty nodes, with *d* the network degree. By considering a special class of network, here tori, we show that more faults can be tolerated for such a routing scenario. More precisely, in this paper we describe a node-to-node torus routing algorithm under the cluster-fault tolerant model: in addition to faulty nodes, faulty clusters of diameter at most one are considered. This furthermore induces new conditions on the number of tolerable faults in such a network, thus refining the conditions stated by Menger’s theorem. And thanks to this cluster-fault tolerant approach, the proposed algorithm is also tolerant to edge faults: given a faulty edge (u,v), it suffices to declare the two nodes *u*, *v* as one faulty cluster.

Given the number of nodes $k^{n}$ and edges $nk^{n}$ involved in an *n*-dimensional *k*-ary torus, solving this cluster-fault tolerant routing problem with a conventional routing algorithm such as Dijkstra’s is clearly impractical: its worst-case time complexity is of polynomial order in the number of nodes or edges of the network, and the same discussion holds with a breadth-first search algorithm [11]. It is thus an effective, and as shown in Section 2 a commonly relied upon approach to describe a routing algorithm for a specific class of graph, here a torus, to provide a practical solution to this routing problem.

As recalled earlier, large sensor networks are nowadays common, and they include thousands of network nodes (i.e., sensor nodes). Fault-tolerance (resilience) and scalability are two key requirements of such networks [12]. This research on the torus interconnection network brings a concrete solution to these issues as detailed below. Indeed, we can rely on the torus network topology to interconnect the sensors, which are themselves attached to “things” of the Internet of things (IoT). In this situation, we assume that these things are static, that is, not moving. In order to report the data collected across the sensor network by each sensor on its thing to the requesting device (the user) which is connected via the Internet to one network node (the sink), the data are delivered through the torus to the requester by forwarding the data according to the paths calculated by the proposed algorithm, paths which connect each sensor node to the (current) network sink.

First, from its definition, and precisely the node degree that does not depend on the network arity, the torus topology provides a high scalability, for instance when compared to other network topologies such as hypercubes [7] and star graphs [13]. Second, considering the large number of sensors involved, faulty sensors are unavoidable. Besides, it is usually expected that the sensor faults take place in a cluster rather than occurring independently. By describing a routing algorithm that is tolerant to faults, the resilience and data transmission performance of the sensor network are increased, which thus offers a higher quality-of-service. Third, with such a connection approach, a distributed sensor network can be easily realised: as mentioned above, a master device accesses the sensor network from the Internet, which further increases the network resilience as none of its nodes has special powers.

In addition to increasing the fault tolerance of data transfers and thus of the whole network, it is important to consider the cluster-fault tolerant model given hardware technical properties of the machinery: it is indeed not rare that faults in such a system occur in clusters. For example, one same electrical unit supplies power to a few nodes. When this power unit fails—battery lifetime is a notorious issue of sensor nodes [14]—the corresponding nodes induce a faulty cluster. As another example, several nodes can be managed by one same hosting or controlling device, like two IoT things which depend on one controlling unit, or a blade inside a cabinet of a supercomputer. Failure of the hosting device would similarly result in a faulty cluster.

**Figure 1:**
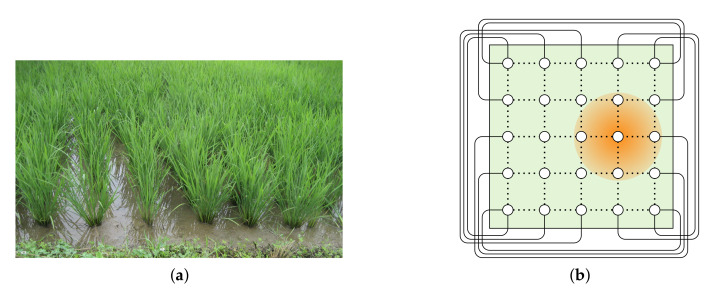
(**a**) A paddy field with young crops. (**b**) A sensor network for a paddy field where the small circles represent sensors. The orange disc represents the transmission range of the sensor at its centre. The wireless and wired interconnection network forms a two-dimensional torus structure.

Smart agriculture is one application field of our research results. For example, in south-east Asian countries paddy fields are popular for the culture of rice. Such paddy fields are wide areas immersed in water (see Figure 1a). The implementation of smart agriculture without interfering with the automated farm operations, a wireless sensor network is suitable to collect information about the paddy field. Sensors in the field are positioned according to the points of a two-dimensional lattice and each sensor has a transmission range so that it can at least communicate with its four neighbour sensors. In addition, the wrap-around edges that connect the sensors placed on the periphery of the paddy field are implemented with wires. Consequently, the sensor network forms a two-dimensional torus structure (see Figure 1b).

Another example with respect to smart agriculture is a plant factory [15,16]. In such a facility, plant pods are organised in a three-dimensional manner and cultivated under optimal growth conditions. In this situation as well, a wireless sensor network is suitable to not interfere with the factory automated operations. Precisely, in the factory the sensors are positioned according to the points of a three-dimensional lattice and each sensor has a transmission range so that it can at least communicate with its six neighbour sensors. In addition, on the floor, walls and ceiling, the wrap-around edges are implemented with wires. As a result, the sensor network forms a three-dimensional torus structure.

The rest of this paper is organised as follows. Previous and related works are discussed in Section 2. Graph notations and definitions together with lemmas and propositions are recalled and established in Section 3. The proposed algorithm is described and exemplified in Section 4. The algorithm correctness is formally established in Section 5; this is a major part of the paper. Complexity analysis, precisely the maximum path length and worst-case time complexity, is formally conducted in Section 6 to evaluate the theoretical performance of the algorithm, and from which the main theorem of this research is induced. Then, in Section 7, the algorithm performance in average is empirically evaluated with computer experimentations and compared to the theoretical values. Finally, this paper is concluded in Section 8.

## 2. Previous and Related Works

The torus topology is often presented as an extension of the mesh network [3] to which “wrap-around edges” have been added and it has been itself further extended [17], for instance to design a hierarchical interconnection network [9].

There exist a few routing algorithms that are tolerant to faults which have been described for a torus network. Torus fault-tolerant routing algorithms based on the simple node-fault tolerant model (i.e., not considering faulty clusters) have been described in [18] with a node-to-node and a node-to-set disjoint paths routing algorithm. In an *n*-dimensional *k*-ary torus (n≥2, k≥4), the former algorithm selects a fault-free path of length at most n⌊k/2⌋+1 in $O(n^{2})$ time with a fault tolerance of at most 2n−1 faulty nodes while the latter algorithm selects *f* (f≤2n) fault-free paths of lengths at most n⌊k/2⌋+1 in $O(n^{3})$ time with a fault tolerance of at most 2n−f faulty nodes. Still based on the simple node-fault tolerant model, an adaptive node-to-node routing algorithm for a 2-dimensional torus has been given in [19].

Other routing algorithms that apply to a torus network have been proposed. A torus set-to-set disjoint paths routing algorithm has been described in [20]. In an *n*-dimensional *k*-ary torus (n≥1, k≥3), this algorithm selects 2n mutually node-disjoint paths between 2n source nodes and 2n destination nodes, without imposing a particular pairing. The paths are selected in $O(kn^{3}+n^{3}\log n)$ time and have lengths that are at most 2(k+1)n. Then, a torus pairwise disjoint paths routing algorithm has been presented in [21]. In an *n*-dimensional *k*-ary torus (n<k, k≥5), given *c* (c≤n) source–destination node pairs, this algorithm finds *c* mutually node-disjoint paths that connect the nodes of the *c* pairs. The paths are selected in $O(nc^{4})$ time and have lengths at most 2k(c−1)+n⌊k/2⌋.

In other networks, fault-tolerance under the simple node-fault tolerant model has been treated, for instance, in hypercubes with a unicast algorithm [22] and a set-to-set disjoint paths routing algorithm [23], in star graphs with a set-to-set disjoint paths routing algorithm [24] and in burnt pancake graphs with a unicast algorithm [25]. Furthermore, fault-tolerant routing with an additional constraint regarding the nodes that can be selected has been discussed, for instance, in hypercubes [26]. Finally, routing algorithms based on the cluster-fault tolerant model have been proposed for burnt pancake graphs with a unicast algorithm [27], hypercubes with a unicast algorithm [28], a node-to-set and set-to-set disjoint paths routing algorithm [29] and a pairwise disjoint paths routing algorithm [30], and star graphs with a unicast algorithm [31] and a node-to-set and pairwise disjoint paths routing algorithm [32], amongst others.

## 3. Preliminaries

First, general graph theory notations and definitions are recalled—the notations and definitions that are not mentioned here are in accordance with [33].

Graphs herein are undirected. For a node *u* in a graph *G*, let $N_{G}\left(u\right)$ be the set of the nodes adjacent to *u* in *G*. A path in a graph *G* is a connected acyclic sub-graph of *G* of maximum degree 2. From this definition, a path necessarily has either one or two nodes of degree at most 1; they are called the end nodes of the path. For the sake of readability, a path is simply denoted by a sequence of nodes and edges as follows: $u_{1}\to u_{2}\to \ldots \to u_{l}$, and is further abbreviated to $u_{1}⇝u_{l}$ when non-ambiguous. The node set V(p) that includes the nodes of a path *p* is simply denoted by *p* when non-ambiguous. Two paths p,q are mutually node-disjoint (or simply “disjoint”) if and only if p∩q=∅. When the path intersection p∩q consists solely of end nodes, the two paths are said to be internally disjoint. A path is fault-free if and only if it does not include a faulty node. A path is blocked if it includes at least one faulty node. The length of a path is its number of edges.

**Definition** **1.** *An n-dimensional k-ary torus T(n,k), n≥1, k≥1 consists of the $k^{n}$ nodes induced by the set ${\left\{0,1,\ldots ,k-1\right\}}^{n}$. A node u of a T(n,k) is thus an n-tuple $\left(u_{1},u_{2},\ldots ,u_{n}\right)$ with $u_{i}$ ($0\leq u_{i}\leq k-1$) the coordinate of u for the dimension i (1≤i≤n). There is an edge between two nodes $u=(u_{1},u_{2},\ldots ,u_{n})$ and $v=(v_{1},v_{2},\ldots ,v_{n})$ of a T(n,k) if and only if ∃j (1≤j≤n) such that ∀i (1≤i≤n, i≠j) $u_{i}=v_{i}$ and $u_{j}=v_{j}\pm 1\;\left(\mathrm{mod}\;k\right)$.*

**Figure 2:**
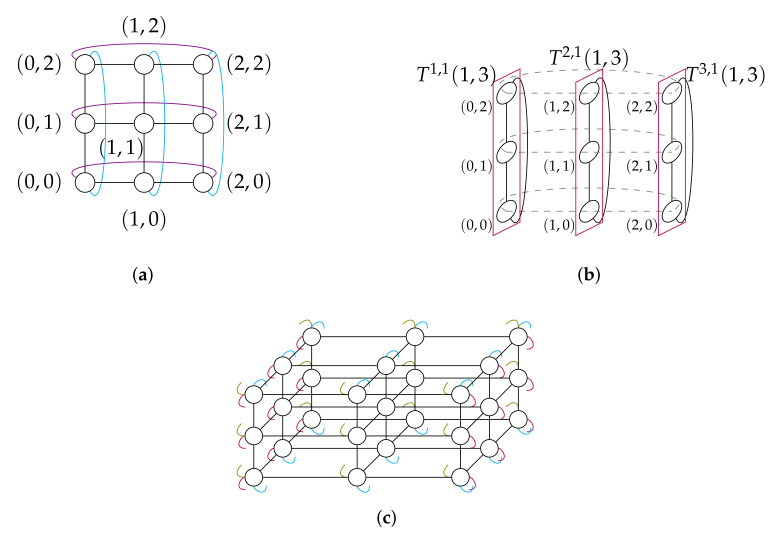
(**a**) A 2-dimensional 3-ary torus T(2,3). (**b**) Illustration of the recursive structure of a torus: a T(2,3) consists in three 1-dimensional 3-ary sub-tori T(1,3). (**c**) A 3-dimensional 3-ary torus T(3,3).

A 2-dimensional 3-ary torus T(2,3) and a 3-dimensional 3-ary torus T(3,3) are illustrated in Figure 2a,c, respectively.

**Definition** **2.** *For a node $u=\left(u_{1},u_{2},\ldots ,u_{n}\right)\in T\left(n,k\right)$ and a dimension δ (1≤δ≤n), define the two paths*

$p_{u,\delta }^{+}=u\to \left(u_{1},u_{2},\ldots ,u_{\delta -1},\left(u_{\delta }+1\right)\;\mathrm{mod}\;k,u_{\delta +1},\ldots ,u_{n}\right)\;\to \left(u_{1},u_{2},\ldots ,u_{\delta -1},\left(u_{\delta }+2\right)\;\mathrm{mod}\;k,u_{\delta +1},\ldots ,u_{n}\right)\to \ldots \;\to \left(u_{1},u_{2},\ldots ,u_{\delta -1},\left(u_{\delta }-2\right)\;\mathrm{mod}\;k,u_{\delta +1},\ldots ,u_{n}\right)\;\;\to \left(u_{1},u_{2},\ldots ,u_{\delta -1},\left(u_{\delta }-1\right)\;\mathrm{mod}\;k,u_{\delta +1},\ldots ,u_{n}\right)$
$p_{u,\delta }^{-}=u\to \left(u_{1},u_{2},\ldots ,u_{\delta -1},\left(u_{\delta }-1\right)\;\mathrm{mod}\;k,u_{\delta +1},\ldots ,u_{n}\right)\;\to \left(u_{1},u_{2},\ldots ,u_{\delta -1},\left(u_{\delta }-2\right)\;\mathrm{mod}\;k,u_{\delta +1},\ldots ,u_{n}\right)\to \ldots \;\to \left(u_{1},u_{2},\ldots ,u_{\delta -1},\left(u_{\delta }+2\right)\;\mathrm{mod}\;k,u_{\delta +1},\ldots ,u_{n}\right)\;\;\to \left(u_{1},u_{2},\ldots ,u_{\delta -1},\left(u_{\delta }+1\right)\;\mathrm{mod}\;k,u_{\delta +1},\ldots ,u_{n}\right)$

In this research, we consider faulty clusters that include at most two nodes. So, a cluster is formally defined as follows.

**Definition** **3.** *A cluster c of a graph G is a connected subgraph of G that is isomorphic either to a $K^{1}$ or to a $K^{2}$, where $K^{n}$ denotes the complete graph of order n. So, a cluster can be denoted simply as a node set.*

**Definition** **4.** *For a graph G and a cluster set C, the set I(G,C)⊆C consists of the clusters of C that have at least one node in G. That is, I(G,C)={c∣c∈C,G∩c≠∅}.*

Next, several torus properties are recalled. First, a torus has a recursive structure; this is Proposition 1 below.

**Proposition** **1.** *Considering a dimension δ (1≤δ≤n), a T(n,k) consists of k sub-tori $T^{i,\delta }\left(n-1,k\right)$ (1≤i≤k). Each sub-torus $T^{i,\delta }\left(n-1,k\right)$ is induced by the $k^{n-1}$ nodes $\left(u_{1},u_{2},\ldots ,u_{\delta -1},i,u_{\delta +1},\ldots ,u_{n}\right)$ of T(n,k) with $u_{j}$ (1≤j≤n,j≠δ) the node coordinate for the dimension j and i that for the dimension δ.*

The three sub-tori $T^{1,1}\left(1,3\right)$, $T^{2,1}\left(1,3\right)$ and $T^{3,1}\left(1,3\right)$ of a T(2,3) induced by δ=1 are shown in Figure 2b.

**Definition** **5.** *For a node u∈T(n,k) and a dimension δ (1≤δ≤n), $T_{u}^{\delta }$ is the sub-torus of u and $t_{u}^{\delta }\in N$ is such that $u\in T^{t_{u}^{\delta },\delta }\left(n-1,k\right)$.*

**Definition** **6.** *For a node $u=\left(u_{1},u_{2},\ldots ,u_{n}\right)\in T\left(n,k\right)$, a dimension δ (1≤δ≤n) and a sub-torus $T^{i,\delta }\left(n-1,k\right)$, $\rho_{u}^{i,\delta }=\left(u_{1},u_{2},\ldots ,u_{\delta -1},i,u_{\delta +1},\ldots ,u_{n}\right)$ corresponds to the node u “projected” into $T^{i,\delta }\left(n-1,k\right)$.*

Second, there exist disjoint paths between sub-tori.

**Lemma** **1.** *For a node u∈T(n,k) and a dimension δ (1≤δ≤n), there exists a set $P_{u}^{i,\delta }$ of 2n−1 internally disjoint paths of lengths at most k−1 between u and the nodes of a sub-torus $T^{i,\delta }\left(n-1,k\right)$ with 1≤i≤k.*

**Proof.**  A constructive proof is given. For the node set $\left\{u_{1},u_{2},\ldots ,u_{2n-2}\right\}=N_{T_{u}^{\delta }}\left(u\right)$, define the path set $P_{u}^{i,\delta }=\left\{\left\{u⇝v\in T^{i,\delta }\left(n-1,k\right)\right\}\subseteq p_{u,\delta }^{+}\right\}\cup {⋃}_{j=1}^{2n-2}\left\{u\to \left\{u_{j}⇝v\in T^{i,\delta }\left(n-1,k\right)\right\}\subseteq p_{u_{j},\delta }^{+}\right\}$. The paths in $P_{u}^{i,\delta }$ are internally disjoint by Definition 2. □

**Lemma** **2.** *For a node u∈T(n,k) and a dimension δ (1≤δ≤n), each path $p\in P_{u}^{i,\delta }$ has a variant $p^{\prime }$ of same end nodes such that the paths of the set $\left(P_{u}^{i,\delta }\setminus P\right)\cup P^{\prime }$ are internally disjoint for any subset $P\subseteq P_{u}^{i,\delta }$ with $P^{\prime }=\left\{p^{\prime }|p\in P\right\}$.*

**Proof.**  A constructive proof is given. For the node set $\left\{u_{1},u_{2},\ldots ,u_{2n-2}\right\}=N_{T_{u}^{\delta }}\left(u\right)$, define ${\tilde{P}}_{u}^{i,\delta }$ a set of path variants with respect to the path set $P_{u}^{i,\delta }$ as ${\tilde{P}}_{u}^{i,\delta }=\left\{\left\{u⇝v\in T^{i,\delta }\left(n-1,k\right)\right\}\subseteq p_{u,\delta }^{-}\right\}\cup {⋃}_{j=1}^{2n-2}\left\{u\to \left\{u_{j}⇝v\in T^{i,\delta }\left(n-1,k\right)\right\}\subseteq p_{u_{j},\delta }^{-}\right\}$. Further define the bijection $r:P_{u}^{i,\delta }\to {\tilde{P}}_{u}^{i,\delta }$ that associates each of the 2n−2 paths $p=u\to u_{j}\to \ldots$ of $P_{u}^{i,\delta }$ to the path $p^{\prime }=u\to u_{j}\to \ldots$ of ${\tilde{P}}_{u}^{i,\delta }$ (1≤j≤2n−2) and the unique path $p=u\to u^{\prime }\notin N_{T_{u}^{\delta }}\left(u\right)\to \ldots$ of $P_{u}^{i,\delta }$ to the path $p^{\prime }=u\to u^{\prime }\notin N_{T_{u}^{\delta }}\left(u\right)\to \ldots$ of ${\tilde{P}}_{u}^{i,\delta }$. The paths in $\left(P_{u}^{i,\delta }\setminus P\right)\cup P^{\prime }$ are internally disjoint for any subset $P\subseteq P_{u}^{i,\delta }$ with $P^{\prime }=\left\{r\left(p\right)|p\in P\right\}$ by Definition 2. □

**Lemma** **3.** *For a node $u=\left(u_{1},u_{2},\ldots ,u_{n}\right)\in T\left(n,k\right)$ and a dimension δ (1≤δ≤n), there exists a set $Q_{u}^{i,\delta }$ of 4n−3 paths of lengths at most k between u and the nodes of a sub-torus $T^{i,\delta }\left(n-1,k\right)$ with 1≤i≤k.*

**Proof.**  A constructive proof is given. For the node set $N={⋃}_{j=1,j\neq \delta }^{n}\left\{\left(u_{1},u_{2},\ldots ,\left(u_{j}+2\right)\;\mathrm{mod}\;k,\ldots ,u_{n}\right),\left(u_{1},u_{2},\ldots ,\left(u_{j}-2\right)\;\mathrm{mod}\;k,\ldots ,u_{n}\right)\right\}$ and the path set $P=\{u\to v\to \left\{w⇝x\in T^{i,\delta }\left(n-1,k\right)\right\}\subseteq p_{w,\delta }^{+}|v\in N_{T_{u}^{\delta }}\left(u\right),w\in N\cap N_{T_{u}^{\delta }}\left(v\right)\}$, define the path set $Q_{u}^{i,\delta }=P_{u}^{i,\delta }\cup P$. □

**Definition** **7.** *For a node u∈T(n,k), a dimension δ (1≤δ≤n), the node set $N={⋃}_{j=1,j\neq \delta }^{n}\left\{\left(u_{1},u_{2},\ldots ,\left(u_{j}+2\right)\;\mathrm{mod}\;k,\ldots ,u_{n}\right),\left(u_{1},u_{2},\ldots ,\left(u_{j}-2\right)\;\mathrm{mod}\;k,\ldots ,u_{n}\right)\right\}$ and the path set $P=\{u\to v\to \left\{w⇝x\in T^{i,\delta }\left(n-1,k\right)\right\}\subseteq p_{w,\delta }^{-}|v\in N_{T_{u}^{\delta }}\left(u\right),w\in N\cap N_{T_{u}^{\delta }}\left(v\right)\}$, define the set of 4n−3 paths ${\tilde{Q}}_{u}^{i,\delta }={\tilde{P}}_{u}^{i,\delta }\cup P$.*

**Figure 3:**
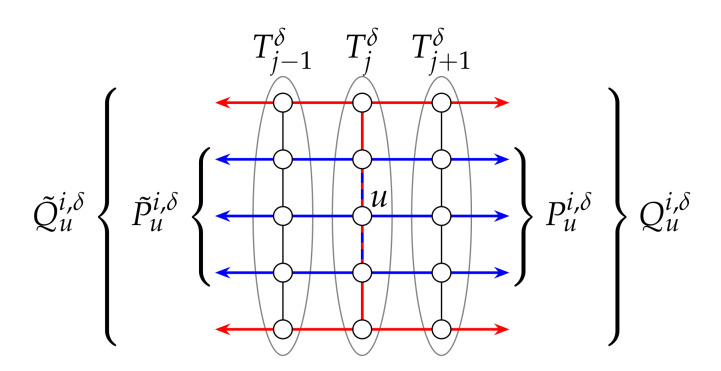
Illustrating the paths of $P_{u}^{i,\delta }$, ${\tilde{P}}_{u}^{i,\delta }$, $Q_{u}^{i,\delta }$ and ${\tilde{Q}}_{u}^{i,\delta }$ in the case of a T(2,5).

The paths of $P_{u}^{i,\delta }$, ${\tilde{P}}_{u}^{i,\delta }$, $Q_{u}^{i,\delta }$ and ${\tilde{Q}}_{u}^{i,\delta }$ are illustrated in the case of a T(2,5) in Figure 3.

## 4. Cluster-Fault Tolerant Routing Algorithm

Inside an *n*-dimensional *k*-ary torus T(n,k) that includes a set *C* of at most 2n−1 faulty clusters (which induce the set of faulty nodes *F*), we describe a routing algorithm that selects a fault-free path between any two nodes s,d∈T(n,k) with s,d∉F.

### 4.1. Algorithm Description

First, we present the assumptions made and the main idea of the proposed routing algorithm. A T(1,k) is isomorphic to a ring, and it is thus trivial to find a fault-free path between any two non-faulty nodes given that there is at most 2n−1=1 faulty cluster. So, we can assume that n≥2.

**Figure 4:**
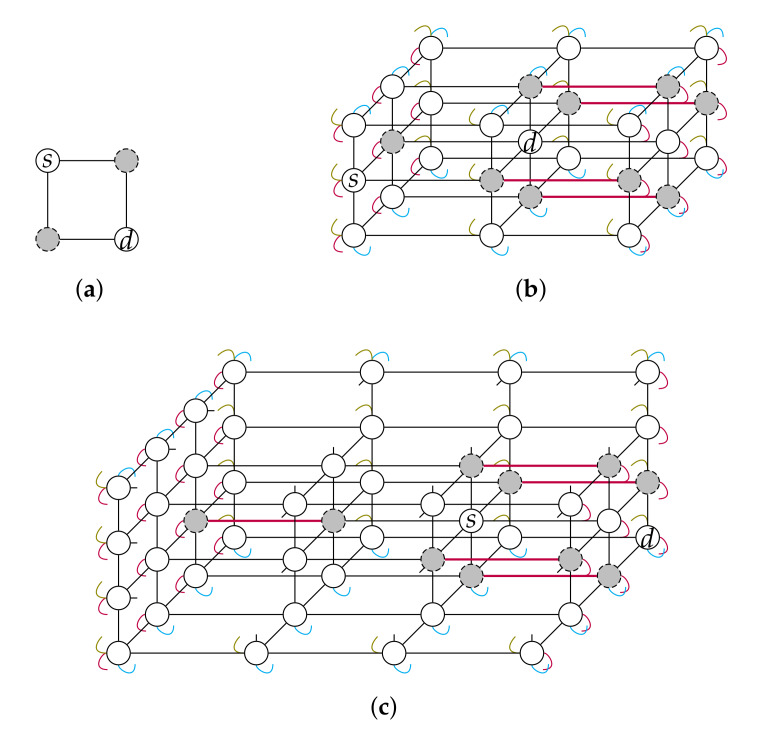
Unsolvable problem instances in the case k=2 (**a**), k=3 (**b**) and k=4 (**c**), within a T(2,2) with two clusters, a T(3,3) with five clusters and a T(3,4) (some nodes are omitted for clarity) with five clusters, respectively. Faulty nodes are greyed and the clusters that include two nodes are materialised with thicker lines.

A T(n,1) has one single node and thus it is trivial to find a fault-free s⇝d path (s=d). It is easy to show that this problem is not solvable when k∈{2,3,4}: see Figure 4. Hence, a torus arity k≥5 is considered hereinafter.

The main idea of this algorithm is to follow a divide-and-conquer approach by routing *s* to a node of $T_{d}^{\delta }$ and to apply this algorithm recursively in $T_{d}^{\delta }$. Consider an arbitrary dimension δ (1≤δ≤n). We distinguish the following mutually exclusive cases.

**Case** **0** (*base case*) T(n,k) is fault-free (i.e., C=F=∅):
  This is simple point-to-point routing. A path between *s* and *d* is selected with a dimension-order routing algorithm [3].
**Case** **1** (*special case*) $T_{d}^{\delta }$ unavailable (i.e., $|I(T_{d}^{\delta },C)|>2n-3$):
  **Case** **1.1** $T_{s}^{\delta }$ unavailable (i.e., $|I(T_{s}^{\delta },C)|>2n-3$):
    We can apply the algorithm recursively in neither $T_{s}^{\delta }$ nor $T_{d}^{\delta }$, so we use another sub-torus. Route *s* and *d* to an available sub-torus $T^{i,\delta }\left(n-1,k\right)$, that is satisfying $|I(T^{i,\delta }\left(n-1,k\right),C)|\leq 2n-3$, with a fault-free path of $P_{s}^{i,\delta }\cup {\tilde{P}}_{s}^{i,\delta }$ and a fault-free path of $P_{d}^{i,\delta }\cup {\tilde{P}}_{d}^{i,\delta }$, respectively.
    Let $p_{s}:s⇝s^{\prime }\in T^{i,\delta }\left(n-1,k\right)$ (resp. $p_{d}:d⇝d^{\prime }\in T^{i,\delta }\left(n-1,k\right)$) be the selected path that connects *s* (resp. *d*) to a node of $T^{i,\delta }\left(n-1,k\right)$. If these paths are not disjoint, consider the node $u\in p_{s}\cap p_{d}$ that is the closest to *s*, discard the sub-paths $\left(u⇝s^{\prime }\right)\subset p_{s}$ and $\left(u⇝d^{\prime }\right)\subset p_{d}$ and terminate. Otherwise, apply this algorithm recursively in $T^{i,\delta }\left(n-1,k\right)$ with $s^{\prime }$ as source node, $d^{\prime }$ as destination node and $\left\{c\cap T^{i,\delta }\left(n-1,k\right)|c\in I\left(T^{i,\delta }\left(n-1,k\right),C\right)\right\}$ as cluster set.
  **Case** **1.2** $T_{s}^{\delta }$ available (i.e., $|I(T_{s}^{\delta },C)|\leq 2n-3$):
    Exchange the roles of *s* and *d*; this is Case 2.
**Case** **2** $T_{d}^{\delta }$ available (i.e., $|I(T_{d}^{\delta },C)|\leq 2n-3$):
  Define $Q=Q_{s}^{t_{d}^{\delta },\delta }\cup {\tilde{Q}}_{s}^{t_{d}^{\delta },\delta }$ the set of 8n−6 paths from *s* to a node of $T_{d}^{\delta }$.
  **Case** **2.1** (*special case*) *s* not routable to $T_{d}^{\delta }$ (i.e., ∀p∈Q,p∩F≠∅):
    Route *s* and *d* to an available sub-torus $T^{i,\delta }\left(n-1,k\right)$, that is satisfying $|I(T^{i,\delta }\left(n-1,k\right),C)|\leq 2n-3$, other than $T_{d}^{\delta }$ with a fault-free path of $P_{s}^{i,\delta }\cup {\tilde{P}}_{s}^{i,\delta }$ and a fault-free path of $P_{d}^{i,\delta }\cup {\tilde{P}}_{d}^{i,\delta }$, respectively.
    Let $p_{s}:s⇝s^{\prime }\in T^{i,\delta }\left(n-1,k\right)$ (resp. $p_{d}:d⇝d^{\prime }\in T^{i,\delta }\left(n-1,k\right)$) be the selected path that connects *s* (resp. *d*) to a node of $T^{i,\delta }\left(n-1,k\right)$. If these paths are not disjoint, consider the node $u\in p_{s}\cap p_{d}$ that is the closest to *s*, discard the sub-paths $\left(u⇝s^{\prime }\right)\subset p_{s}$ and $\left(u⇝d^{\prime }\right)\subset p_{d}$ and terminate. Otherwise, apply this algorithm recursively in $T^{i,\delta }\left(n-1,k\right)$ with $s^{\prime }$ as source node, $d^{\prime }$ as destination node and $\left\{c\cap T^{i,\delta }\left(n-1,k\right)|c\in I\left(T^{i,\delta }\left(n-1,k\right),C\right)\right\}$ as cluster set.
  **Case** **2.2** (*general case*) *s* routable to $T_{d}^{\delta }$ (i.e., ∃p∈Q, p∩F=∅):
    Route *s* to $T_{d}^{\delta }$ with a fault-free path of *Q*. Let $p_{s}:s⇝s^{\prime }\in T_{d}^{\delta }$ be the selected path of *Q* that connects *s* to a node of $T_{d}^{\delta }$. If $d\in p_{s}$ (i.e., $s^{\prime }=d$), terminate. Otherwise, apply this algorithm recursively in $T_{d}^{\delta }$ with $s^{\prime }$ as source node, *d* as destination node and $\left\{c\cap T_{d}^{\delta }|c\in I\left(T_{d}^{\delta },C\right)\right\}$ as cluster set.

### 4.2. Routing Example in a T(3,5)

We give a non-trivial example of execution trace for the proposed routing algorithm. Let s=(1,1,0), d=(1,0,1) and C={{(1,1,1),(0,1,1)},{(0,1,0)},{(1,0,0)},{(2,1,0)},{(1,2,0),(1,2,4)}} be the source node, destination node and cluster set in a T(3,5), respectively. Furthermore, δ is arbitrarily initialised to 3. The execution trace of the algorithm is given in Table 1. The leftmost column indicates the iteration step, and the “Sub-Torus” column the sub-torus selected for recursion. It is recalled that the torus arity (here, k=5) is constant throughout the execution of the algorithm; it is thus not repeated in the table. The first iteration step falls in Case 2.1, the second in Case 2.2 and the third in Case 0 of the algorithm.

## 5. Proof of Correctness

In this section, the correctness of the proposed algorithm is established. Each of all the distinguished cases is treated separately.

### 5.1. Case 0

There is no faulty node inside T(n,k), so a dimension-order routing algorithm can be applied.

### 5.2. Case 1.1

The sub-tori $T_{s}^{\delta }$ and $T_{d}^{\delta }$ each include at least 2n−2 clusters (possibly partially, i.e., $|I(T_{d}^{\delta },C)|>2n-3$) and are thus unavailable in order to solve the problem recursively. Here are two necessary conditions for this situation to occur: 1) $T_{s}^{\delta }$ is adjacent to $T_{d}^{\delta }$ (i.e., $t_{d}^{\delta }=t_{s}^{\delta }\pm 1\;\left(\mathrm{mod}\;k\right)$) and 2) out of the at most 2n−1 clusters, at least 2n−3 of them have two nodes, with one node in $T_{s}^{\delta }$ and the other in $T_{d}^{\delta }$.

**Figure 5:**
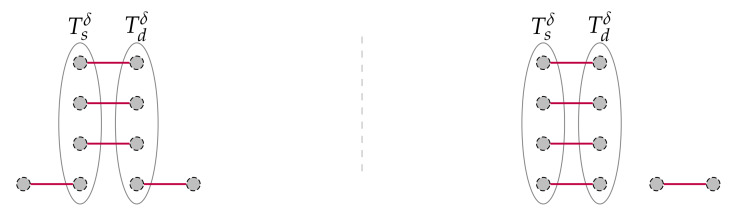
The two possible cluster repartitions in Case 1.1 with respect to $T_{s}^{\delta }$ and $T_{d}^{\delta }$ when n=3 and thus at most five clusters. Ellipses separate sub-tori.

First, we show that there exist at least three sub-tori that are available for recursion, that is, that include at most 2n−3 clusters. It is recalled that both $T_{s}^{\delta }$ and $T_{d}^{\delta }$ are not. At least 2n−3 clusters are included (completely contained) in $T_{s}^{\delta }\cup T_{d}^{\delta }$. Hence, for both $T_{s}^{\delta }$ and $T_{d}^{\delta }$ to satisfy $|I(T_{d}^{\delta },C)|>2n-3$, there remains at most two faulty nodes that are included in other sub-tori (i.e., neither in $T_{s}^{\delta }$ nor $T_{d}^{\delta }$). These two faulty nodes are either part of the same cluster, or, they are part of two distinct 2-node clusters that have one node in $T_{s}^{\delta }$ and one node in $T_{d}^{\delta }$, respectively. So, importantly, if these at most two faulty nodes are part of two distinct 2-node clusters, these two faulty nodes are necessarily located in different sub-tori. See Figure 5.

To apply the algorithm recursively inside a sub-torus, it can include at most 2n−3 clusters. So, one sub-torus can be made unavailable with at least 2n−2 clusters. Therefore, either the at most one 2-node cluster or the at most two faulty nodes located in distinct sub-tori suffice not to make another sub-torus unavailable for recursion since they would induce at most one cluster inside a sub-torus, and 1<2n−2 given that n≥2. Therefore, since there exist at least k≥5 sub-tori and at most two of them are unavailable ($T_{s}^{\delta }$ and $T_{d}^{\delta }$), at least three sub-tori always remain available for recursion.

Next, we show that both *s* and *d* are routable to at least one of these three available sub-tori. If *s* and *d* are adjacent, select s→d and there is nothing else to prove. So, we can assume that *s* and *d* are not adjacent. We distinguish the following mutually exclusive sub-cases which are exhaustive.

**Figure 6:**
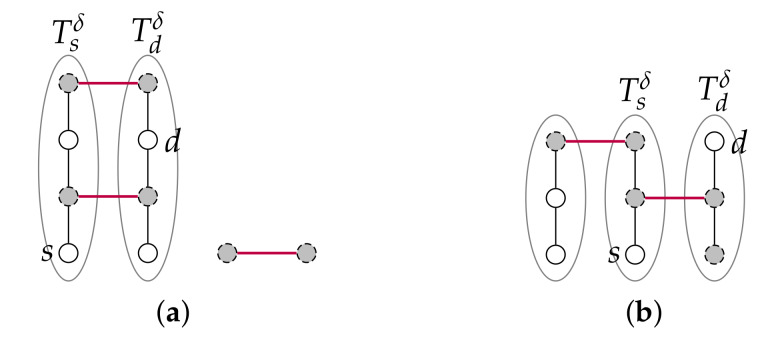
The two situations for Case 1.1’s sub-case $N_{T_{s}^{\delta }}\left(s\right)\cup N_{T_{d}^{\delta }}\left(d\right)\subseteq F$. (**a**) k=4 required; (**b**) k=3 required. Ellipses separate sub-tori, faulty nodes are greyed and the clusters that include two nodes are materialised with thicker lines.

*Sub-case $N_{T_{s}^{\delta }}\left(s\right)\cup N_{T_{d}^{\delta }}\left(d\right)\subseteq F$.* This case can occur only when n=2 and it implies that either (a) there are two 2-node clusters each included in $N_{T_{s}^{\delta }}\left(s\right)\cup N_{T_{d}^{\delta }}\left(d\right)$, or (b) there is only one such cluster and the other two clusters respectively have at least one node in $N_{T_{s}^{\delta }}\left(s\right)$ and one node in $N_{T_{d}^{\delta }}\left(d\right)$. As shown in Figure 6, the former case (a) occurs only when k=4 and the latter case (b) only when k=3. Hence, given that k≥5 is assumed, these two cases shall never occur and there is thus nothing to prove.

*Sub-case $N_{T_{s}^{\delta }}\left(s\right)\subset F$ and $N_{T_{d}^{\delta }}\left(d\right)\neg \subset F$ (the case $N_{T_{s}^{\delta }}\left(s\right)\neg \subset F$ and $N_{T_{d}^{\delta }}\left(d\right)\subset F$ is discussed similarly).* Since both $T_{s}^{\delta }$ and $T_{d}^{\delta }$ unavailable, at least 2n−3 clusters each have one node in $T_{s}^{\delta }$ and the other in $T_{d}^{\delta }$, and either (a) at least one other cluster also has, or (b) the two other clusters each have at least one node in $T_{s}^{\delta }\cup T_{d}^{\delta }$. In the former case (a), at least 2n−2 clusters each have one node in $T_{s}^{\delta }$ and the other in $T_{d}^{\delta }$, and those clusters thus can block at most one path of $\left\{p_{s,\delta }^{+},p_{s,\delta }^{-}\right\}$ and at most one path of $\left\{p_{d,\delta }^{+},p_{d,\delta }^{-}\right\}$. The remaining cluster, if any (there is at most 1), can either block at most two paths of $\left\{p_{s,\delta }^{+},p_{s,\delta }^{-}\right\}$ for at most two available sub-tori, or at most two paths of $\left\{p_{d,\delta }^{+},p_{d,\delta }^{-}\right\}$ for at most two available sub-tori. Since both situations cannot occur at the same time, there always remains at least one available sub-torus to which both *s* and *d* are routable. In the latter case (b), 2n−3 clusters cannot block any path of $\left\{p_{s,\delta }^{+},p_{s,\delta }^{-},p_{d,\delta }^{+},p_{d,\delta }^{-}\right\}$ for the same reason, and the other two clusters each have at least one node in $T_{s}^{\delta }\cup T_{d}^{\delta }$, hence they can block at most two paths of $\left\{p_{s,\delta }^{+},p_{s,\delta }^{-}\right\}$ for at most one available sub-torus, and at most two paths of $\left\{p_{d,\delta }^{+},p_{d,\delta }^{-}\right\}$ for at most one available sub-torus (the blocked at most two sub-tori are necessarily distinct). Therefore, there always remains at least one available sub-torus to which both *s* and *d* are routable.

*Sub-case $N_{T_{s}^{\delta }}\left(s\right)\neg \subset F$ and $N_{T_{d}^{\delta }}\left(d\right)\neg \subset F$.* This is the same proof as for the previous case (i.e., $N_{T_{s}^{\delta }}\left(s\right)\subset F$ and $N_{T_{d}^{\delta }}\left(d\right)\neg \subset F$).

### 5.3. Case 1.2

Case 2 is applied, so refer to the proofs of Cases 2.1 and 2.2.

### 5.4. Case 2.1

**Figure 7:**
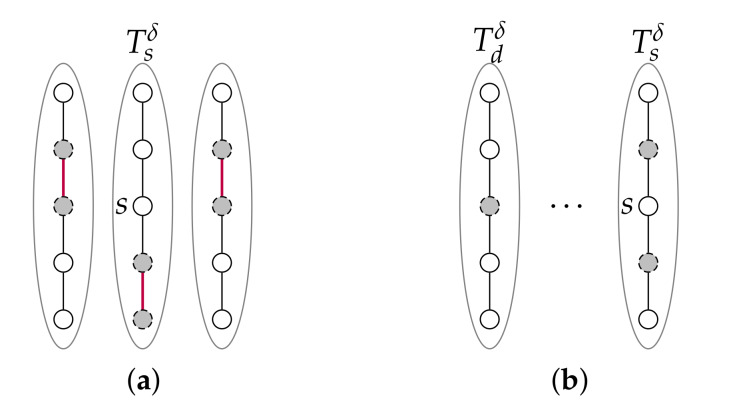
Situations of Case 2 in a T(2,5): (**a**) the situation in Case 2.2 where the paths of $P_{s}^{t_{d}^{\delta },\delta }\cup {\tilde{P}}_{s}^{t_{d}^{\delta },\delta }$ do not suffice to route *s* to another sub-torus; (**b**) the situation in Case 2.1 where *s* is not routable to $T_{d}^{\delta }$.

We indeed need to consider the paths of $Q_{s}^{t_{d}^{\delta },\delta }\cup {\tilde{Q}}_{s}^{t_{d}^{\delta },\delta }$ (they are each of length at most *k*) given that the paths of $P_{s}^{t_{d}^{\delta },\delta }\cup {\tilde{P}}_{s}^{t_{d}^{\delta },\delta }$ (they are each of length at most k−1) may not suffice to route *s* to another sub-torus as shown in Figure 7a.

So, *s* not routable to $T_{d}^{\delta }$ implies that the 8n−6 paths of $Q=Q_{s}^{t_{d}^{\delta },\delta }\cup {\tilde{Q}}_{s}^{t_{d}^{\delta },\delta }$ are all blocked. This situation occurs only when both 1) at least 2n−2 clusters each have at least one node in $N_{T_{s}^{\delta }}\left(s\right)\cup N_{T_{d}^{\delta }}\left(\rho_{s}^{t_{d}^{\delta },\delta }\right)$, and 2) one of these clusters includes $\rho_{s}^{t_{d}^{\delta },\delta }$, or there is one other cluster with no node in $T_{s}^{\delta }\cup N_{T_{d}^{\delta }}\left(\rho_{s}^{t_{d}^{\delta },\delta }\right)$ that does. See Figure 7b.

We show the existence of a sub-torus available for recursion other than $T_{d}^{\delta }$. Since at least 2n−2 clusters each including at least one node in $N_{T_{s}^{\delta }}\left(s\right)\cup N_{T_{d}^{\delta }}\left(\rho_{s}^{t_{d}^{\delta },\delta }\right)$, they can induce at most two unavailable sub-tori. If two unavailable sub-tori are so induced, they are $T_{s}^{\delta }$ and one sub-torus adjacent to $T_{s}^{\delta }$ (that is not $T_{d}^{\delta }$ since $T_{d}^{\delta }$ available by assumption), and the remaining cluster includes $\rho_{s}^{t_{d}^{\delta },\delta }$ and thus suffices not to make one additional sub-torus unavailable. If only one unavailable sub-torus is so induced, it is necessarily $T_{s}^{\delta }$ since $T_{d}^{\delta }$ available by assumption, and thus exactly 2n−2 clusters each have at least one node in $N_{T_{s}^{\delta }}\left(s\right)$ and the remaining cluster which has no node in $T_{s}$ includes $\rho_{s}^{t_{d}^{\delta },\delta }$. Therefore, since there exist at least k≥5 sub-tori and at most two of them are unavailable (one of which necessarily being $T_{s}^{\delta }$), at least three sub-tori always remain available for recursion.

Next, we show that both *s* and *d* are routable to at least one of these three available sub-tori. If *s* and *d* are adjacent, select s→d and there is nothing else to prove. So, we can assume that *s* and *d* are not adjacent. We distinguish the following mutually exclusive sub-cases which are exhaustive.

*Sub-case $N_{T_{s}}\left(s\right)\subset F$.* Exactly 2n−2 clusters thus have at least one node in $T_{s}^{\delta }$ ($T_{s}^{\delta }$ is thus unavailable). These clusters cannot block a path of $\left\{p_{s,\delta }^{+},p_{s,\delta }^{-}\right\}$ and can block at most two paths of $\left\{p_{d,\delta }^{+},p_{d,\delta }^{-}\right\}$ for at most one available sub-torus. The remaining cluster necessarily includes $\rho_{s}^{t_{d}^{\delta },\delta }$ and can thus block at most two paths of $\left\{p_{s,\delta }^{+},p_{s,\delta }^{-}\right\}$ for at most two available sub-tori, one being $T_{d}^{\delta }$; it can block no path of $\left\{p_{d,\delta }^{+},p_{d,\delta }^{-}\right\}$. Therefore, there always remains at least one available sub-torus other than $T_{d}^{\delta }$ to which both *s* and *d* are routable.

*Sub-case $N_{T_{s}}\left(s\right)⊄F$.* Since $T_{d}^{\delta }$ available, $N_{T_{d}}\left(d\right)⊄F$. Thus, there exist two non-faulty nodes $u\in N_{T_{s}^{\delta }}\left(s\right)$ and $v\in N_{T_{d}}\left(d\right)$. If the remaining cluster (i.e., that includes no node of $T_{s}^{\delta }\cup N_{T_{d}^{\delta }}\left(\rho_{s}^{t_{d}^{\delta },\delta }\right)$) includes $\rho_{s}^{t_{d}^{\delta },\delta }$, it can block at most two paths of $\left\{p_{s,\delta }^{+},p_{s,\delta }^{-}\right\}$ for at most one available sub-torus. If it does not, it can block either: at most two paths of $\left\{p_{s,\delta }^{+},p_{s,\delta }^{-}\right\}$ for at most two available sub-tori, or at most two paths of $\left\{p_{d,\delta }^{+},p_{d,\delta }^{-}\right\}$ for at most two available sub-tori, or at most two paths of $\left\{p_{s,\delta }^{+},p_{s,\delta }^{-}\right\}$ and at most two paths of $\left\{p_{d,\delta }^{+},p_{d,\delta }^{-}\right\}$ for at most one available sub-torus. Therefore, there always remains at least one available sub-torus other than $T_{d}^{\delta }$ to which both *s* and *d* are routable.

### 5.5. Case 2.2

There is nothing to show given that $T_{d}^{\delta }$ is available for recursion and *s* is routable to $T_{d}^{\delta }$.

## 6. Complexity Analysis

In this section, we establish the length of a longest path as selected by the proposed algorithm, as well as the worst-case time complexity of this algorithm. It is assumed that the value of each dimension of a node address can be accessed in constant time O(1). Let τ(c,n) be the worst-cast time complexity of the algorithm when applied in a T(n,k) with c≤2n−1 faulty clusters, and let λ(c,n) be the maximum length of the generated path.

**Case** **0** The path is obtained with a dimension-order routing algorithm. Hence, in a T(n,k) it is of length at most n⌊k/2⌋. This algorithm takes O(nk) time.
**Case** **1** It takes O(n) time to check if $|I(T_{d}^{\delta },C)|>2n-3$ holds.
  **Case** **1.1** It takes O(n) time to check if $|I(T_{s}^{\delta },C)|>2n-3$ holds. An available sub-torus $T^{i,\delta }\left(n-1,k\right)$ is found when $|I(T^{i,\delta }\left(n-1,k\right),C)|\leq 2n-3$ holds and both s,d are routable to it with the defined paths. Checking for one sub-torus candidate for $T^{i,\delta }\left(n-1,k\right)$ whether $|I(T^{i,\delta }\left(n-1,k\right),C)|\leq 2n-3$ holds takes O(n) time. The path $p_{s}$ for one sub-torus candidate for $T^{i,\delta }\left(n-1,k\right)$ can be found in O(nk|F|) time since by Lemma 2$|P_{s}^{i,\delta }\cup {\tilde{P}}_{s}^{i,\delta }|=4n-2$ and with each path from those tried for $p_{s}$ being of length at most k−1 by Lemma 1. A similar discussion holds for $p_{d}$. So, checking if one sub-torus is suitable as $T^{i,\delta }\left(n-1,k\right)$ takes O(nk|F|) time. Hence, an available sub-torus $T^{i,\delta }\left(n-1,k\right)$ can be found in $O(nk^{2}|F|)$ by enumerating the *k* sub-tori. Checking the intersection of $p_{s}$ and $p_{d}$ takes $O(k^{2})$ time. If it is not empty, sub-paths are discarded in constant time and the algorithm is terminated. Otherwise, the algorithm is applied recursively in $T^{i,\delta }\left(n-1,k\right)$, thus inducing a $\tau (c^{\prime },n-1)$ time complexity and a $\lambda (c^{\prime },n-1)$ maximum path length, with $c^{\prime }\leq 2\left(n-1\right)-1$ (and obviously $c^{\prime }\leq c$). So, in total, this case is $O(nk^{2}|F|+\tau \left(c^{\prime },n-1\right))$ time and induces a $2k-2+\lambda (c^{\prime },n-1)$ maximum path length.
  **Case** **1.2** The time complexity and maximum path length induced by Case 2 apply.
**Case** **2** It is not needed to check again whether $T_{d}^{\delta }$ is available.
  **Case** **2.1** It takes O(n) time to check whether *s* is routable to $T_{d}^{\delta }$. An available sub-torus $T^{i,\delta }\left(n-1,k\right)$ is found when $|I(T^{i,\delta }\left(n-1,k\right),C)|\leq 2n-3$ holds and both s,d are routable to it with the defined paths. Checking for one sub-torus candidate for $T^{i,\delta }\left(n-1,k\right)$ whether $|I(T^{i,\delta }\left(n-1,k\right),C)|\leq 2n-3$ holds takes O(n) time. The path $p_{s}$ for one sub-torus candidate for $T^{i,\delta }\left(n-1,k\right)$ can be found in O(nk|F|) time since by Lemma 2$|P_{s}^{i,\delta }\cup {\tilde{P}}_{s}^{i,\delta }|=4n-2$ and with each path from those tried for $p_{s}$ being of length at most k−1 by Lemma 1. A similar discussion holds for $p_{d}$. So, checking if one sub-torus is suitable as $T^{i,\delta }\left(n-1,k\right)$ takes O(nk|F|) time. Hence, an available sub-torus $T^{i,\delta }\left(n-1,k\right)$ can be found in $O(nk^{2}|F|)$ by enumerating the k−1 sub-tori ($T_{d}^{\delta }$ excluded). Checking the intersection of $p_{s}$ and $p_{d}$ takes $O(k^{2})$ time. If it is not empty, sub-paths are discarded in constant time and the algorithm is terminated. Otherwise, the algorithm is applied recursively in $T^{i,\delta }\left(n-1,k\right)$, thus inducing a $\tau (c^{\prime },n-1)$ time complexity and a $\lambda (c^{\prime },n-1)$ maximum path length, with once again $c^{\prime }\leq 2\left(n-1\right)-1$. So, in total, this case is $O(nk^{2}|F|+\tau \left(c^{\prime },n-1\right))$ time and induces a $2k-2+\lambda (c^{\prime },n-1)$ maximum path length.
  **Case** **2.2** The path $p_{s}$ can be found in O(nk|F|) time since by Lemma 3 and Definition 7$|Q_{s}^{t_{d}^{\delta },\delta }\cup {\tilde{Q}}_{s}^{t_{d}^{\delta },\delta }|=8n-6$ and with each path from those tried for $p_{s}$ being of length at most *k* by Lemma 3. The algorithm is applied recursively in $T_{d}^{\delta }$, thus inducing a $\tau (c^{\prime },n-1)$ time complexity and a $\lambda (c^{\prime },n-1)$ maximum path length, with once again $c^{\prime }\leq 2\left(n-1\right)-1$. So, in total, this case is $O(nk|F|+\tau \left(c^{\prime },n-1\right))$ time and induces a $k+\lambda (c^{\prime },n-1)$ maximum path length.

From this discussion, we can derive the following theorem.

**Theorem** **1.** *In a T(n,k) with k≥5, given two fault-free nodes s,d and a set of at most 2n−1 faulty clusters, a fault-free path between s and d of length at most n(2k+⌊k/2⌋−2) can be found in $O(n^{2}k^{2}|F|)$ time with F the set of faulty nodes induced by the faulty clusters.*

**Proof.**  The existence of a fault-free path s⇝d is shown in Section 4 and Section 5. The complexities are derived from Section 6 which induces the following recursive expressions regarding the time complexity and maximum path length:

$$\begin{array}{cc} \tau (0,n) & =O(nk)\\ \tau (c,n) & =O(nk^{2}|F|+\tau \left(c^{\prime },n-1\right))\;\mathrm{if}c>0 \end{array}$$

and

$$\begin{array}{cc} \lambda (0,n) & =n\lfloor k/2\rfloor \\ \lambda (c,n) & =2k-2+\lambda (c^{\prime },n-1)\;\mathrm{if}c>0 \end{array}$$

with $c^{\prime }\leq 2\left(n-1\right)-1$ (and obviously $c^{\prime }\leq c$). The relation $c^{\prime }\leq 2\left(n-1\right)-1$ is the invariant of the recursion. Since *n* is decreased by one at each step, $c^{\prime }$ is guaranteed to reach 0, that is the base case of the recursion. Therefore, the total worst-case time complexity of the proposed algorithm is $O(n^{2}k^{2}|F|)$ and the maximum path length is n(2k−2)+n⌊k/2⌋=n(2k+⌊k/2⌋−2). □

The described algorithm selects a fault-free path of length at most n(2k+⌊k/2⌋−2) with an $O(n^{2}k^{2}|F|)$ worst-case time complexity with *F* the set of faulty nodes induced by the faulty clusters. The maximum path length is of the same order as the network diameter: O(nk), which is thus on par with previous works on node-to-node routing under the cluster-fault tolerant model [27,28,31].

## 7. Empirical Evaluation

Now that the worst-case complexities have been established in Section 6, we inspect the average behaviour of the proposed algorithm, implemented to this end. Two experiments were conducted: the first one aims at measuring the maximum length of a path selected by the proposed algorithm, and the second one at measuring the average execution time taken by the algorithm to solve one instance of the torus cluster-fault tolerant routing problem. These experiments were conducted on a computer equipped with an Intel Core i5-1035G7 processor (clocked at 1.20 GHz) and 8 GB RAM, and running Windows 10 Home 64-bit.

**Figure 8:**
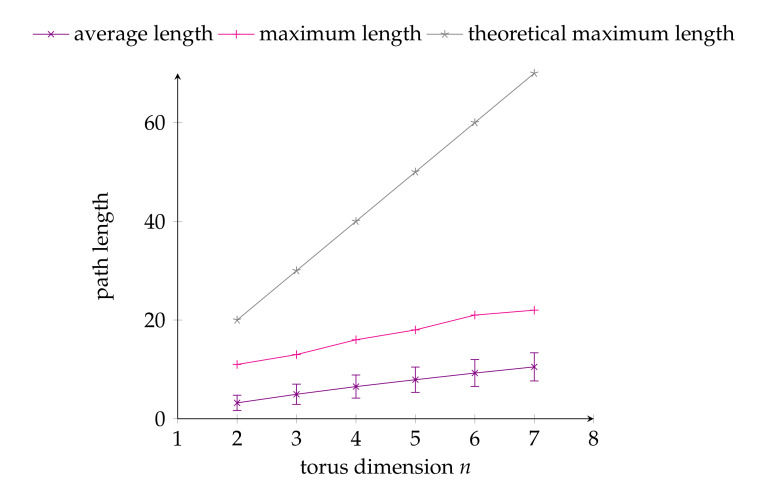
Empirical evaluation: average and maximum (with standard deviation) path lengths of the paths selected by the proposed algorithm in a T(n,5).

The experimental conditions for the first experiment (i.e., maximum path length measurement) were as follows: in a T(n,5), the source node and destination nodes are randomly selected in the set of all the torus nodes. The torus arity *k* was fixed to 5 in this experiment to maximize the routing difficulty as indeed the number of faults depends on *n* and not on *k*. Then, the maximum number of faulty clusters 2n−1 that can be tolerated were also randomly generated. The faulty clusters are all of diameter one to once again maximize the routing problem difficulty (i.e., a higher number of faulty nodes). Then, the algorithm implementation was used to solve the corresponding routing problem and the length of the selected path output was recorded. This process was repeated 10,000 times for each value of *n* with 2≤n≤7, each time calculating two path length values: the maximum path length and the average path length of the 10,000 selected paths. The results of this first experiment are shown in Figure 8, together with the theoretical maximum path length as established previously in Section 6 for reference.

**Figure 9:**
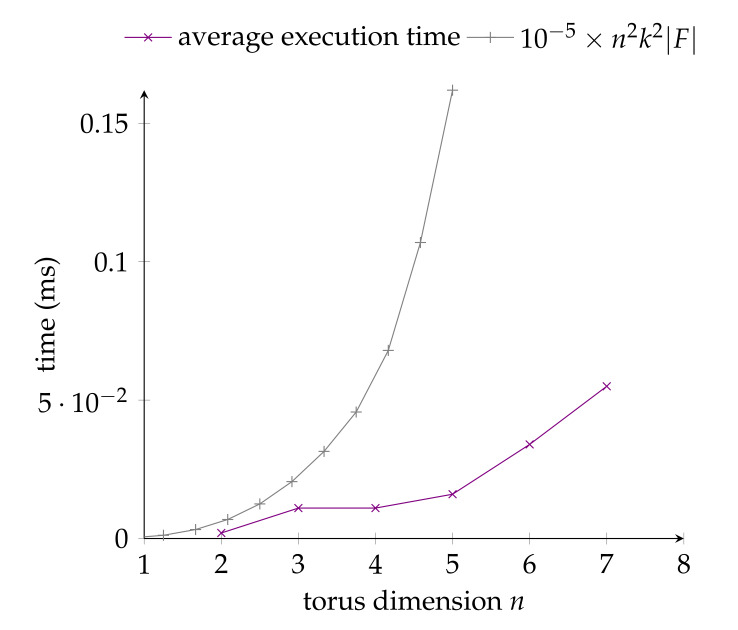
Empirical evaluation: average execution time to solve one problem instance with the proposed algorithm in a T(n,max{5,n+1}).

The second experiment (i.e., execution time measurement) was conducted in the same experimental conditions as the first experiment at the exception that the routing problem was solved in a T(n,max{5,n+1}): the arity *k* was set to max{5,n+1} in this time experiment to evaluate the average time complexity as *k* and *n* both increase. The path selection algorithm was run 10,000 times for each (n,max{5,n+1}) pair with 2≤n≤7, each time measuring the real CPU time (i.e., excluding the time for garbage collection) taken to solve the problem instance. The obtained results are given in Figure 9, together with the worst-case time complexity as established previously in Section 6 for reference.

The following observations can be made from the obtained experimental results. First, regarding the maximum path length, one can note that it remains at distance from the theoretical upper bound, which is an indicator of the good performance of the algorithm. Second, regarding the average execution time, one can note that it remains well below the worst-case time complexity, which is yet another indicator of the efficiency of the proposed algorithm.

## 8. Conclusions

The growing number of Internet-connected devices and their sensors, comparable to that of computing nodes included in modern supercomputers, induces large interconnection networks. Hence, the performance of networks on this scale is tied to efficient and robust data routing. For example, major supercomputer makers such as IBM, Cray and Fujitsu have been relying on the torus topology for the interconnection network for its advantageous topological properties. The torus topology is also applicable to interconnect sensor networks, for instance, to report information collected by sensors across the network to the network user. Given the huge number of network nodes involved, faults are very likely to occur. A routing algorithm in a torus that is tolerant to faults is thus key for the future of such networks and has direct implications to the quality-of-service issue by reducing the number of failed data communications. Furthermore, hardware technical properties inducing that faults often happen in clusters (e.g., a same power supply unit applies to a few nodes), it is critical to not only tolerate node faults but also cluster-faults. Improving on Menger’s condition on the maximum number of node faults that can be tolerated, and on torus fault-tolerant routing algorithms described in previous works, we have proposed in this paper for the first time a node-to-node routing algorithm in a torus that is tolerant to cluster-faults. In a T(n,k) with at most 2n−1 faulty clusters of diameter at most 1, the described algorithm selects a fault-free path of length at most n(2k+⌊k/2⌋−2) with an $O(n^{2}k^{2}|F|)$ worst-case time complexity with *F* the set of faulty nodes induced by the faulty clusters.

Regarding future works, it will be meaningful to first try to consider faulty clusters of diameter 2, possibly reducing the number of tolerated faulty clusters. Then, selecting several fault-free disjoint paths between the source and destination nodes can be considered. Furthermore, measuring the average performance of the proposed algorithm and comparing the results with the formally established worst-case complexities (maximum path length and time complexity) is yet another research route.

## Figures and Tables

**Table 1:** A sample execution trace of the algorithm for a non-trivial routing example in a T(3,5).

⥀	δ	*n*	*s*	*d*	*C*	Sub-Torus	Selected Paths
*1*	3	3	(1,1,0)	(1,0,1)	{{(1,1,1),(0,1,1)}, { (0, 1, 0) }, { (1, 0, 0) }, { (2, 1, 0) }, { (1, 2, 0), (1, 2, 4) } }	$T^{4,3}\left(2,5\right)$	$s=\left(1,1,0\right)\to \left(1,1,4\right)=s^{\prime }$ $d=\left(1,0,1\right)\to \left(1,0,2\right)\;\to \left(1,0,3\right)\to \left(1,0,4\right)=d^{\prime }$
*2*	2	2	(1,1,4)	(1,0,4)	{{(1,2,4)}}	$T^{0,2}\left(1,5\right)$	$s=\left(1,1,4\right)\to \left(1,0,4\right)=s^{\prime }$
*3*	-	1	(1,0,4)	(1,0,4)	∅	-	s=(1,0,4)=d
*Output*: s=(1,1,0)→(1,1,4)→(1,0,4)→(1,0,3)→(1,0,2)→(1,0,1)=d							

## References

[1] Hsu C.L., Lin J.C.C. (2016). An empirical examination of consumer adoption of Internet of things services: Network externalities and concern for information privacy perspectives. Comput. Hum. Behav..

[2] Nordrum A. (2016). Popular Internet of things forecast of 50 billion devices by 2020 is outdated. IEEE Spectrum.

[3] Duato J., Yalamanchili S., Ni L. (2003). Interconnection Networks: An Engineering Approach.

[4] Cray Inc (2010). Cray XE6 Brochure. https://www.cray.com/sites/default/files/resources/CrayXE6Brochure.pdf.

[5] Ajima Y., Inoue T., Hiramoto S., Uno S., Sumimoto S., Miura K., Shida N., Kawashima T., Okamoto T., Moriyama O. Tofu interconnect 2: System-on-chip integration of high-performance interconnect. Proceedings of the 29th International Supercomputing Conference.

[6] TOP500 (2017). TOP500 List Refreshed, US Edged out of Third Place. https://www.top500.org/news/top500-list-refreshed-us-edged-out-of-third-place/.

[7] Saad Y., Schultz M. (1988). Topological properties of hypercubes. IEEE Trans. Comput..

[8] Seitz C. (1985). The cosmic cube. Commun. ACM.

[9] Bossard A., Kaneko K. (2015). Torus-Connected Cycles: A simple and scalable topology for interconnection networks. Int. J. Appl. Math. Comput. Sci..

[10] Menger K. (1927). Zur allgemeinen Kurventheorie. Fundam. Math..

[11] Sedgewick R. (2002). Algorithms in C—Part 5, Graph Algorithms.

[12] Chakraborty S., Chakraborty S., Nandi S., Karmakar S. (2015). Fault resilience in sensor networks: Distributed node-disjoint multi-path multi-sink forwarding. J. Netw. Comput. Appl..

[13] Akers S., Krishnamurthy B. (1989). A group-theoretic model for symmetric interconnection networks. IEEE Trans. Comput..

[14] Guerroumi M., Pathan A.S.K. (2018). Hybrid data dissemination protocol (HDDP) for wireless sensor networks. Wirel. Netw..

[15] Shi X., An X., Zhao Q., Liu H., Xia L., Sun X., Guo Y. (2019). State-of-the-art Internet of things in protected agriculture. Sensors.

[16] Ohishi-Yamazaki M., Watanabe M., Nakanishi A., Che J., Horiuchi N., Ogiwara I. (2018). Shortening of the juvenile phase of the southern highbush blueberry (*Vaccinium corymbosum* L. interspecific hybrid) grown controlled rooms under artificial light. Hortic. J..

[17] Yang Y., Funahashi A., Jouraku A., Nishi H., Amano H., Sueyoshi T. (2001). Recursive diagonal torus: An interconnection network for massively parallel computers. IEEE Trans. Parallel Distrib. Syst..

[18] Gu Q.P., Peng S. (1996). Fault tolerant routing in toroidal networks. IEICE Trans. Inf. Syst..

[19] Li Y., Peng S., Chu W. Online adaptive fault-tolerant routing in 2D torus. Proceedings of the Third International Symposium on Parallel and Distributed Processing and Applications.

[20] Kaneko K., Bossard A. (2017). A set-to-set disjoint paths routing algorithm in tori. Int. J. Netw. Comput..

[21] Bossard A., Kaneko K. (2018). Torus pairwise disjoint-path routing. Sensors.

[22] Gu Q.P., Peng S. (1999). Unicast in hypercubes with large number of faulty nodes. IEEE Trans. Parallel Distrib. Syst..

[23] Gu Q.P., Okawa S., Peng S. (1996). Set-to-set fault tolerant routing in hypercubes. IEICE Trans. Fundam. Electron. Commun. Comput. Sci..

[24] Gu Q.P., Peng S. (1996). Set-to-set fault tolerant routing in star graphs. IEICE Trans. Inf. Syst..

[25] Iwasaki T., Kaneko K. (2010). Fault-tolerant routing in burnt pancake graphs. Inf. Process. Lett..

[26] Bossard A., Kaneko K. (2015). Hypercube fault tolerant routing with bit constraint. Int. J. Netw. Comput..

[27] Iwasawa N., Watanabe T., Iwasaki T., Kaneko K. Cluster-fault-tolerant routing in burnt pancake graphs. Proceedings of the 10th International Conference on Algorithms and Architectures for Parallel Processing.

[28] Gu Q.P., Peng S. (1996). An efficient algorithm for node-to-node routing in hypercubes with faulty clusters. Comput. J..

[29] Gu Q.P., Peng S. (1998). Node-to-set and set-to-set cluster fault tolerant routing in hypercubes. Parallel Comput..

[30] Gu Q.P., Peng S. (1997). *k*-pairwise cluster fault tolerant routing in hypercubes. IEEE Trans. Comput..

[31] Gu Q.P., Peng S. (1995). Node-to-node cluster fault tolerant routing in star graphs. Inf. Process. Lett..

[32] Gu Q.P., Peng S. (2000). Cluster fault-tolerant routing in star graphs. Networks.

[33] Diestel R. (2010). Graph Theory.

